# Methods for Detecting Suspicious Information From Individual Transactions of Pharmaceutical Products via Twitter (now X): Retrospective Observational Study

**DOI:** 10.2196/91103

**Published:** 2026-06-19

**Authors:** Ruoyu Zhang, Kazuko Kimura, Naoko Yoshida

**Affiliations:** 1 Kanazawa University Kanazawa, Ishikawa Japan; 2 Society for Medicines Security Research Kanazawa, Ishikawa Japan; 3 Okayama University Okayama, Okayama Japan

**Keywords:** online pharmaceuticals, social networking services, text mining, machine learning, decision-tree analysis

## Abstract

**Background:**

Individual transactions involving pharmaceutical products via social networking service (SNS) are considered an inappropriate distribution route and may serve as a guise for illicit business-to-consumer activities. In Japan, individual transactions of pharmaceutical products via the internet are recognized as inappropriate distribution routes, which not only lead to the inappropriate use of pharmaceutical products but also require more active monitoring and guidance from the viewpoint of pharmaceutical security and quality assurance.

**Objective:**

This study aimed to develop a method to accurately detect SNS tweets suspected of involving individual transactions of pharmaceutical products, using text data from Twitter (subsequently rebranded as X), the primary platform for such activities in Japan.

**Methods:**

We applied text mining to 1389 text tweets suspected of involving individual pharmaceutical transactions. Using the hashtag “#Okusuri mogumogu,” which was identified through manual searching and is commonly associated with trading psychotropic pharmaceuticals, we collected 7499 tweets posted in 2022 and 6461 tweets posted from January 1 to March 31, 2023, using our web crawler program. After manually categorizing whether each tweet was related to individual pharmaceutical transactions, we extracted words and summarized their occurrences and frequencies using the 2022 dataset. A decision tree model was then generated using the 2022 dataset and validated using the 2023 dataset to evaluate the reliability of detecting transaction-related tweets.

**Results:**

Using web crawling, the number of tweets identified using the hashtag “#Okusuri mogumogu” was 7499 in 2022 and 6461 in the first 3 months of 2023. The crawling results also showed that the number of detectable tweets increased closer to the crawl date, suggesting that SNS tweets may frequently be deleted. From 3228 extracted words in the 2022 dataset, 452 were significantly associated with tweets suspected of involving individual transactions. Highly indicative terms included “kyuu” (request), “yuzuri” (transfer), “DM” (direct message), and transaction-related hashtags. The chi-square automatic interaction detection model demonstrated stable discriminative performance (area under the receiver operating characteristic curve values: training 0.83 and 0.84; Gini coefficient: training 0.65 and test 0.68). The overall accuracy using the 2023 validation dataset was 82.31%, indicating reasonable generalizability despite linguistic fragmentation and the presence of partial word forms characteristic of Japanese text.

**Conclusions:**

Using transaction-related tags, text mining, and machine learning, we identified key terms linked to individual pharmaceutical transactions and developed a predictive model. This approach may aid in preventing inappropriate online transactions of pharmaceutical products.

## Introduction

Social media platforms, or social networking services (SNSs), are a medium for communicating with others through the internet and are widely used daily. The number of social media users worldwide is projected to increase from 4.59 billion in 2022 to 6.03 billion in 2028, and the number of social media users in Japan is projected to increase from 102 million in 2022 to 113 million in 2027 [1]. Among the SNS platforms most widely used in Japan, the third most popular is Twitter (subsequently rebranded as X), with 66.58 million users [2].

SNS platforms can be used to send out information about oneself, obtain the latest or important information, and even purchase or obtain certain items. In recent years, pharmaceutical E-commerce platforms and online pharmacies have increased, new formats have emerged, and online sales of medicines have become increasingly dynamic. This means that there has been a gradual transfer of people’s acquisition of medicines from offline to online. Especially during the COVID-19 pandemic, some situations worldwide make it difficult to go to a health care facility in person to obtain long-term medicines, which has led to a rapid growth in online sales of medicines [3-5].

However, not all patients have access to medicines on the internet through formal sources. In addition to some countries and regions lacking relevant laws and regulations that lead to the unregulated purchase of pharmaceuticals [5], some people access online pharmaceutical products for improper purposes, such as overdosing. Some buyers wish to purchase medicines anonymously, deliberately avoiding formal sources of pharmaceutical products. Fraudulent sellers can take advantage of these situations. Because of its convenience, there is an overabundance of information on the internet that makes improper individual transactions difficult to regulate, and social networking sites have become a breeding ground for some inappropriate transactions. Although common sense should preclude engaging in illegal activities, some individuals prioritize profit and engage in activities that violate the law. There may also be cases where individuals violate the law without knowing that they are doing so. One such case is the unauthorized sale of pharmaceutical products, and we have confirmed that some psychotropic medications are being bought and sold on SNS platforms [6,7]. Such individual transactions represent inappropriate distribution channels that may conceal business-to-consumer activities and pose public health risks.

In Japan, the sale of pharmaceutical products is regulated under the Pharmaceutical and Medical Device Act, which requires appropriate licensing for distribution. Unauthorized sales via SNS sites therefore constitute regulatory violations and present ongoing challenges for pharmaceutical governance and quality assurance.

To address these regulatory challenges, regulatory authorities and platform operators often rely on manual surveillance, keyword-based searches, and user reporting systems to identify suspicious content. In academic research, automated detection methods based on natural language processing and machine learning have been increasingly applied to social media data to identify drug abuse–related discussions, counterfeit drug promotion, and illicit online pharmacy advertisements. Recent studies have further developed machine learning and deep learning–based frameworks for automated detection of relevant content on social media platforms, demonstrating the feasibility and improved performance of such approaches for analyzing health-related discussions and harms using large-scale social media datasets [6,7]. However, the focus of these studies has not been on individual-to-individual transactions of legally approved pharmaceutical products. A recent review also summarized applications, challenges, and methodological trends in pharmacovigilance using social media data [8].

Despite these advances, relatively limited research has specifically focused on unauthorized peer-to-peer transactions of legally manufactured pharmaceutical products within regulated markets such as Japan, representing an important research gap. Among past studies, several limitations remain. First, keyword-based monitoring is prone to both false positives and false negatives because users frequently use abbreviations, euphemisms, or coded language when referring to pharmaceutical products [9,10]. Second, previous studies have primarily focused on detecting discussions of drug misuse or illegal online pharmacy advertising; systematic investigation of individual-to-individual pharmaceutical transactions on SNS platforms is scarce. Furthermore, the generalizability and operational feasibility of these models for routine regulatory monitoring have not always been sufficiently evaluated.

To help strengthen the monitoring of pharmaceutical transactions subject to control to help prevent health hazards to the public, we aimed to develop a method for accurately detecting SNS tweets that are suspected of involving individual transactions for pharmaceutical products using text data from X, the main SNS platform used for these transactions in Japan. In recent years, web crawling and text mining techniques have been increasingly applied to social media data to detect illegal or inappropriate activities. Web crawling is a method in which a web crawler program follows links on the internet to visit websites and duplicate and store information on web pages. This method is used in various fields to collect and use information that appears online. If the web crawler is archiving websites, it copies and saves information as it goes. The archives are usually stored in such a way that they can be viewed, read, and navigated as if they were live on the web, but they are preserved as “snapshots” [11]. Text mining, text data mining, or text analytics is the process of deriving high-quality information from text [12]. Additionally, the chi-square test is used in the analysis of contingency tables when the sample size is large. In simpler terms, this test is primarily used to examine whether two categorical variables (two dimensions of the contingency table) independently influence the test statistic (values within the table) [13]. As the amount of health care megadata continues to grow, increasingly more researchers are using machine learning, which is capable of extracting important and relevant information hidden within massive amounts of data and making useful predictions based on this information [14-19]. A decision tree is a machine learning technique that identifies clusters of data in which certain features often appear and that generates classification rules for these clusters. In the present study, for holdout validation, all data were randomly divided into training data and test data to evaluate the generalization performance of the model. The analysis method used was chi-square automatic interaction detection (CHAID), which is a type of decision tree analysis used to build a decision tree model based on the chi-square test and *F* test. In the field of pharmacy, decision trees can be used to identify and predict adverse drug reactions [20], medication adherence [21], drug abuse [22], and pharmacy services [23], among others.

Building upon these analytical frameworks, in this study, we aimed to develop a surveillance method for accurately detecting SNS tweets suspected of involving individual pharmaceutical transactions using text data from Twitter.

## Methods

### Web Crawling and Data Defined

**Figure 1:**
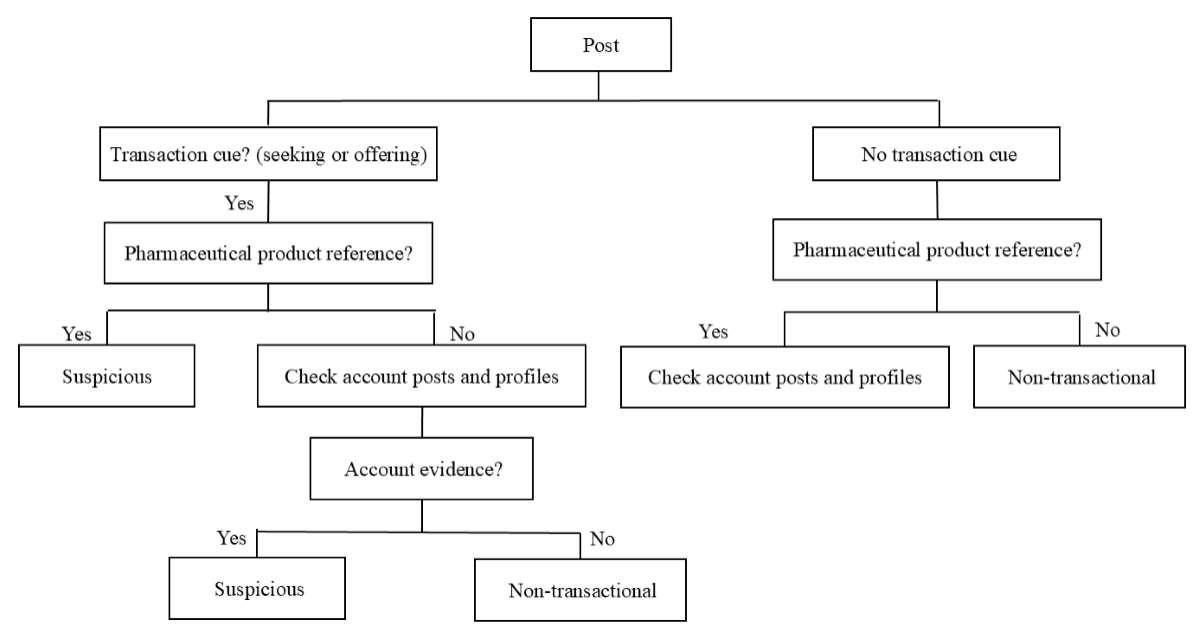
Structured decision framework for manual classification of tweets suspected of involving pharmaceutical transaction tweets.

This study was conducted after receiving approval for academic research access to the Twitter application programming interface (API; admission date: November 29, 2022). The keywords used in our search included the hashtag “#Okusuri mogumogu,” which is often used for trading in psychotropic pharmaceuticals. We analyzed tweets during 2012-2022 related to the trading of pharmaceuticals between individuals and, through manual searching, identified the hashtag “#Okusuri mogumogu” as a keyword for data collection. We developed a web crawler program (IBM Japan, Tokyo, Japan) for this study and collected information on tweets made and not deleted between January 1, 2022, at 0:00 AM JST and March 31, 2023, at 23:59 PM JST. Tweet data collected via web crawling on Twitter were divided into 2 datasets, one in 2022 and a second in 2023. Each tweet was manually reviewed and classified according to its content and account characteristics to determine whether it was suspected of involving an individual pharmaceutical transaction. The manual classification was based on predefined rule-based criteria developed prior to analysis to ensure systematic and reproducible identification of suspicious tweets. Given the contextual and evolving nature of language used in online pharmaceutical transactions, classification required interpretative assessment beyond the presence of isolated keywords. Accordingly, classification was conducted using a structured framework incorporating multiple indicators of transactional intent. These indicators included: (1) the presence of transaction- related expressions commonly used in Japanese SNS contexts, such as “seeking” (求), indicating a request to obtain pharmaceuticals, or “offering” (譲), indicating willingness to provide these; (2) the mention of pharmaceutical-related terms, including formal drug names, abbreviations, slang expressions, emojis, or drug-related images; (3) indications of contact transfer, such as requests to move to direct messaging or other external platforms; and (4) account-level characteristics suggesting repeated similar tweets or explicit transactional intent in the profile description. The predefined indicators served as structured guidance for classification; however, final determinations were made based on a holistic evaluation of the tweet content and contextual account information rather than reliance on any single indicator. The classification procedure is illustrated schematically in Figure 1.

In accordance with the annotation framework illustrated in Figure 1, 2 reviewers (NY and KK) with expertise in pharmaceutical regulation independently conducted an initial pilot annotation using a randomly selected subset of tweets to refine the classification criteria and ensure consistent interpretation. Interreviewer agreement was assessed to confirm sufficient consistency prior to full-scale annotation. After the agreement was deemed acceptable, the remaining tweets were annotated by one reviewer (ZR) based on the finalized criteria. Ambiguous cases were discussed as needed to maintain consistency. The manually assigned classification (1=suspected transaction; 0=non-transactional) was included in the dataset. Additionally, statistically significant keywords identified in prior text mining analysis were coded as binary variables, indicating the presence “1” or absence “0” of each keyword within a tweet.

All data collection procedures were conducted under approved academic API access, and the study protocol received ethics committee approval, as described below.

### Keyword Extraction: Text Mining

Individual word cloud extraction was performed using MATLAB Text Analytics Toolbox (MathWorks Inc). We did not include words appearing fewer than four times so as to eliminate words with a low frequency. This process allowed us to obtain all words in the collected tweets that could be related to individual pharmaceutical transactions, as well as the number of occurrences of each word.

In the course of our investigation, we noted that a number of terms used in SNS tweets (including words that could not be successfully extracted but that appeared in the text of tweets) were created in response to searches by SNS officials like those from Twitter as well as relevant monitoring services, conducted to determine whether users are engaged in individual transactions that may be in violation of the law, such as using emojis or Kanji characters to represent pharmaceutical products being sold. In fact, our text mining program successfully extracted emojis that were used to refer to pharmaceutical products being bought or sold, or words used in these transactions after processing the emojis in a way that allowed them to be recognized.

### Analysis: Word Feature Extraction

To obtain more definitive information on keywords suspected of being related to individual pharmaceutical transactions, an appropriate analysis method is needed. We therefore used our two datasets to conduct the following analyses.

### Statistical Analysis

In this research, we used SPSS (version 19.0.0; SPSS Inc) to statistically analyze associations between the frequencies of each word and tweets suspected of involving individual pharmaceutical transactions. We considered *P* values of <.05 to indicate statistically significant probability. To account for multiple comparisons across all tested words, the Benjamini-Hochberg false discovery rate correction was applied. Only words with *P* values of <.01 after this correction are presented in Table S1 in Multimedia Appendix 1.

### Feature Prediction: Machine Learning

To extract feature words as well, we used the decision tree approach to extract keywords that have correlations with individual transactions involving pharmaceutical products to help guide future surveillance efforts. Decision tree analysis was performed using data mining software (IBM SPSS Modeler version 18.3; IBM Japan). All word-level binary variables derived from the 2022 dataset were entered into the CHAID algorithm without prior manual feature selection. Variable selection occurred internally during tree construction based on chi-square–based node splitting.

In this study, the 2022 dataset was randomly partitioned prior to model construction. For internal evaluation, training:test ratios of 7:3 and training:test:validation ratios of 6:2:2 were applied to assess model stability and performance. Tweets were assigned to partitions without overlap. The final ensemble model, comprising 10 component CHAID trees, was trained exclusively on the 2022 dataset. To prevent overfitting, the maximum tree depth was limited to five levels, and the minimum number of cases required for parent and child nodes was set to 2% and 1% of the total sample size, respectively. Tree growth was terminated when no further statistically significant splits were identified, using a significance level of 0.05 with Bonferroni adjustment. Predictions from the ensemble were generated by aggregating the outputs of the individual component models.

Model validation was then performed using a temporally independent dataset from 2023. Tweets were assigned to datasets strictly based on the posting date to ensure no overlap with the training data. The 2023 dataset was not involved in feature selection, parameter tuning, model training, or ensemble optimization, and was reserved solely for post hoc evaluation. This approach ensured that the validation process was independent and that information from the training set could not influence the assessment of model performance.

### Ethical Considerations

This study was conducted using publicly available social media data collected via academic access to the Twitter API, which was formally approved for academic research (approval date: November 29, 2022). The study protocol was reviewed and approved by the Medical Ethics Committee of Kanazawa University (approval number 2025-466 115205). All analyses were performed using publicly available data, and no attempts were made to identify individual users. The study adhered to Twitter’s terms of service and API usage policies. Ethical considerations related to surveillance monitoring and data privacy were incorporated throughout the research process.

## Results

### Web Crawling and Data Defined

**Figure 2:**
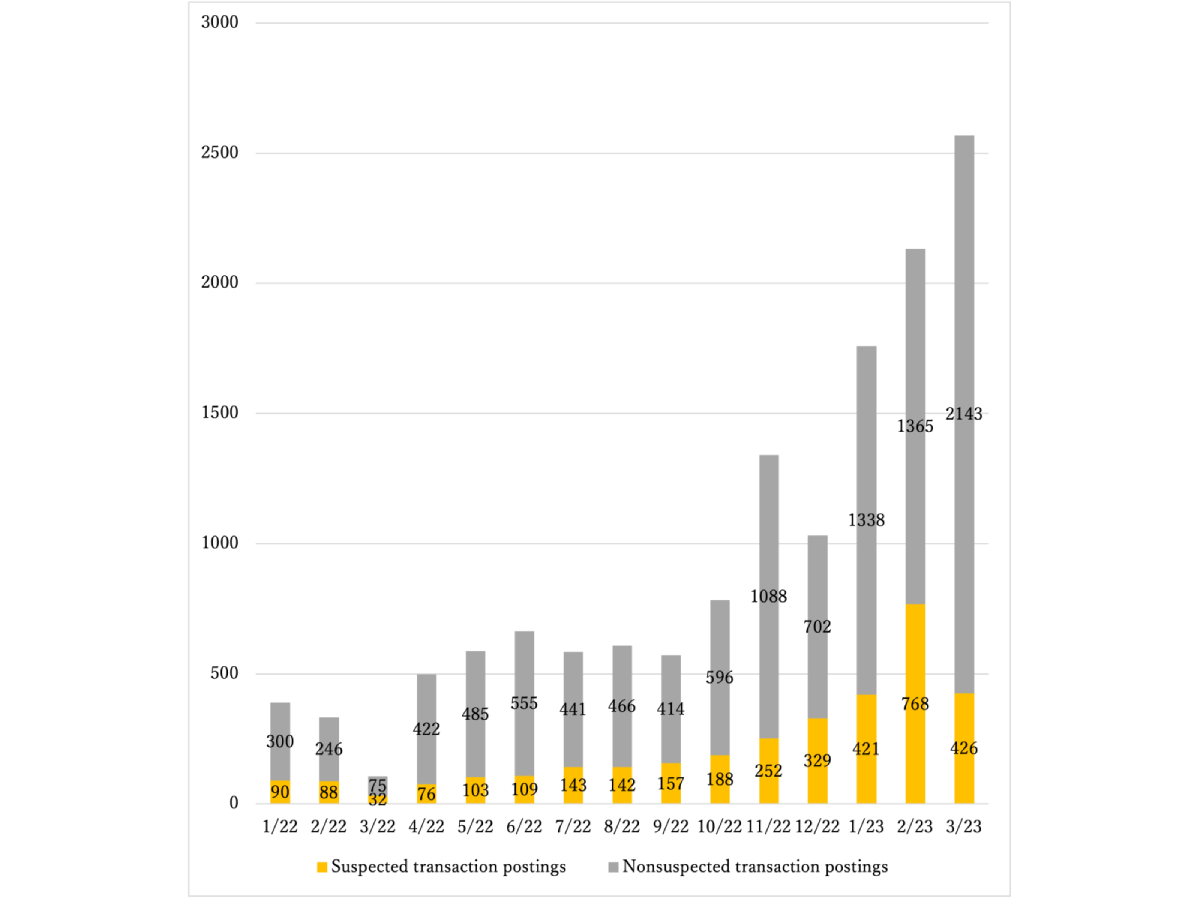
Monthly total of tweets. X-axis represents month/year, and Y-axis represents the number of tweets.

We conducted a manual search of the 2 datasets using the keywords anabolic steroids, testosterone, oxymetholone, and methandienone [9]. We did not find any tweets on Twitter related to individual transactions for these pharmaceuticals. Therefore, we examined whether tweets involving these types of transactions used specific pharmaceutical product names (including commercial product names); we found it important to target certain keywords to detect correlated tweets. The number of tweets identified using the hashtag “#Okusuri mogumogu” in the year 2022 was 7499; this number in the first 3 months of 2023 was 6461. Analysis showed that the number of tweets that could be identified, as well as tweets identified that were related to individual transactions, both tended to increase closer to the time of our data collection (Figure 2).

We found that the closer our search was to the web crawler run date, the more tweets were identified, suggesting that it may be difficult to collect information from SNS tweets because these can be deleted at any time.

### Separation of Transactions and Word Counts

We defined each tweet according to whether its content was suspected of being related to individual transactions involving pharmaceuticals. In 2023, 1625 (25.15%) tweets were classified as suspected individual transaction tweets, and 4836 (74.85%) tweets were not related to individual transactions. The total number of words extracted from the collected tweets was 3228 in 2022 and 2866 in 2023. A cross-tabulation of the 3228 words extracted from 2022 tweets revealed 452 words that were significantly associated with the presence or absence of individual transactions involving pharmaceutical products in each tweet, as well as the occurrence of each word (Table 1).

Black squares are accounts and information that cannot be disclosed.

In the translation process, we used Hiragana, Katakana, Kanji, and English case correspondences. Because all three expressions are recognized as different characters in Japanese and therefore recognized as different keywords by the text mining program used, the same word appears several times in the table, for case differentiation only.

Kanji characters are uppercase first, Katakana characters are all uppercase, Hiragana characters are lowercase, and English characters are extracted as is.

Each word was ranked according to the proportion of tweets in which it was used in relation to such individual transactions. The most frequently appearing term in tweets potentially related to individual pharmaceutical transactions was #1 “kyuu” (the word for “request” in Japanese), and 81 of 83 (97.59%) tweets in which “kyuu” was used were related to individual transactions for pharmaceutical products. The second most common word was #2 “yuzuri” (the word for “give” or “transfer” in Japanese). Of 103 tweets in which this word was used, 100 (97.09%) were related to individual transactions for pharmaceutical products. The next most frequently extracted words were: #3 “pushcart,” #4 receive,” #5 “profile,” #6 “yuzutt” (like “givin” in English), #7 “list,” #8 “customary,” #9 “enlightenment,” and #10 “requestion,” all of which were closely related to pharmaceutical transactions. The hashtag “#Okusuri mogumogu” was ranked 293rd by our web crawler. Of the total 6682 tweets in which this word was used, 1641 (24.56%) tweets involved individual transactions for pharmaceuticals.

**Table 1:** Top 10 rankings of extracted and statistically significant words from the 2022 collected tweets list.

Ranking numbers	Extracted words (Japanese)	Total number of tweets in which the word appears (α)^a^	Total number of tweets in which the word appears among tweets with suspected transactions (β)	Percentage of β/α
1	Kyuu (求)	83	81	97.59%
2	Yuzuri (譲り)	103	100	97.09%
3	Pushcart (取引)	28	27	96.43%
4	receive (頂ける)	25	24	96%
5	profile (プロファイル)	23	22	95.65%
6	yuzutt (譲っ)	131	125	95.42%
7	List (リスト)	42	40	95.24%
8	customary (恒例)	21	20	95.24%
9	enlightenment (啓蒙)	20	19	95%
10	requestion (依頼)	19	18	94.74%

^a^Total of 452 words, sorted according to the total number of tweets in which the word appears among tweets with suspected transactions=β/ Total number of tweets in which the word appears =α, in descending order; *P*<.01 for all words.

We have added the split English words where the Japanese characters are split, and we have added the original words that may have been split in the (*) sections as an annotation reference.

### Classification and Forecasting Models: Word Extraction

All 7499 tweets in 2022 were manually classified as suspected of being individual transactions involving pharmaceutical products, or “1” (n=1709), or not suspected of being individual transactions involving pharmaceutical products, or “0” (n=5790), as the objective variables. The ratio of training: testing data were adjusted to create a CHAID-based model to predict and classify tweets. As a result, word combinations that characterized tweets related to individual transactions in pharmaceuticals were extracted.

**Figure 3:**
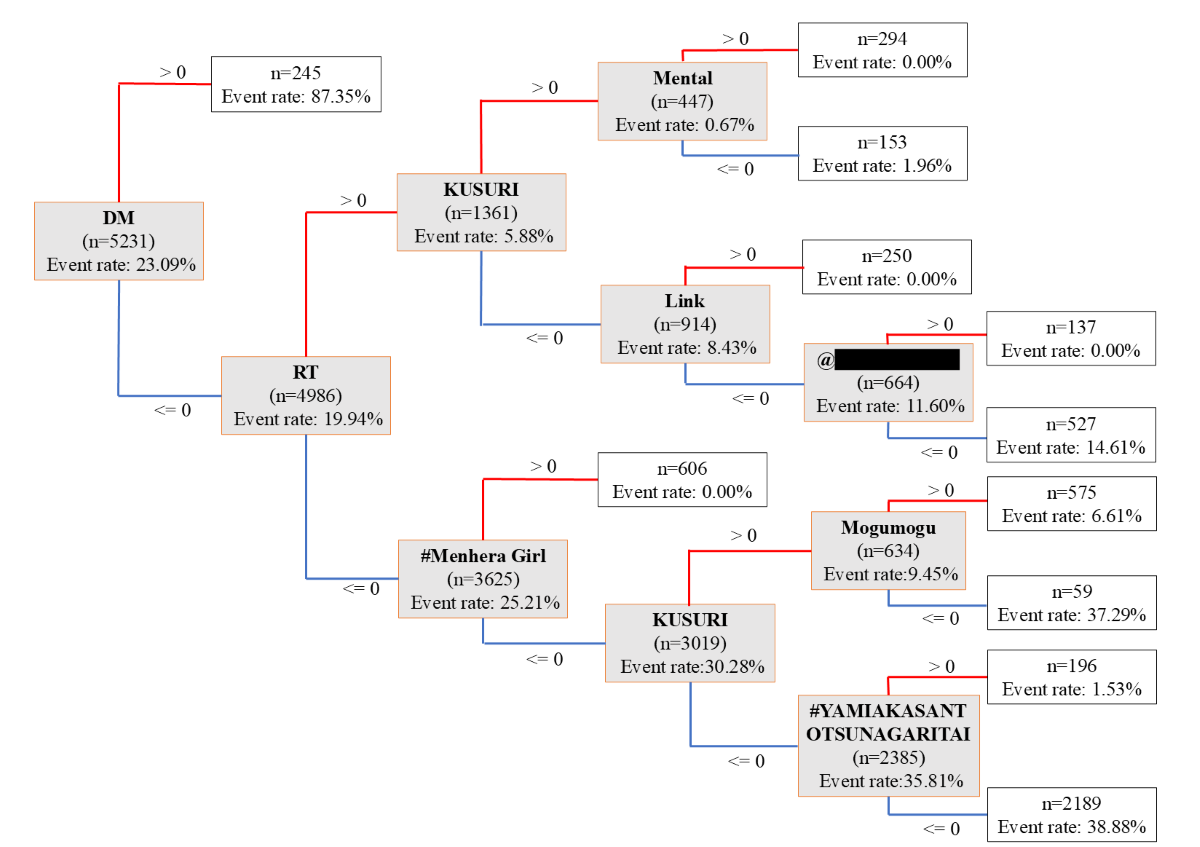
Decision tree model generated from the collection of submissions for the year 2022 (training:testing=7:3) (0): tweets and the percentage in which the word appears in tweets not related to transactions (1): number and percentage of tweets in which the word appears among tweets with suspected transactions.

In the testing stage, we found that using ratios of 7:3 and 6:2:2 each for results involving correct and incorrect model decisions and evaluations were not very different, and the 7:3 model was observed to be cleaner. Therefore, considering that there was not much content in the data, using the part that was divided out for verification was not as effective as using this part for learning and testing. Eventually, we chose the 7:3 ratio to develop the model. The following terms were extracted as predictor variables, in order of importance: “KUSURI” (drugs in Japanese; can also be extracted from Okusuri mogumogu), “DM” (direct message), “#Menhera Girl,” “RT” (retweet), “#YAMIAKASANTOTSUNAGARITAI” (*I Want To Connect With Sick Account Users in Japanese), “Mental,” “#Okusuri moguru,” and “Link” (Figure 3).

The term “DM” was extracted in the first level of the tree as one of the words characteristically used in tweets related to individual transactions for pharmaceutical products (Figure 3). We noted a pattern in the characteristics of words used in tweets suspected of being pharmaceutical transactions: inclusion of the term “DM” had a significantly higher rate of involving individual transactions for pharmaceutical products (1208/5231, 23.10%; *P*<.001, chi-square test). This was denoted as “1” according to our definition for such tweets (as opposed to “0,” meaning not involving individual transactions for pharmaceutical products). The accuracy of the model was checked; the accuracy rate was 80.41% for the training data and 82.32% for the test data, with 4206 correctly categorized tweets and 1025 miscategorized tweets (19.59%). As for test data, the model reached an accuracy rate of 82.32% with 1867 correctly categorized tweets and 401 miss tweets (17.68%).

**Figure 4:**
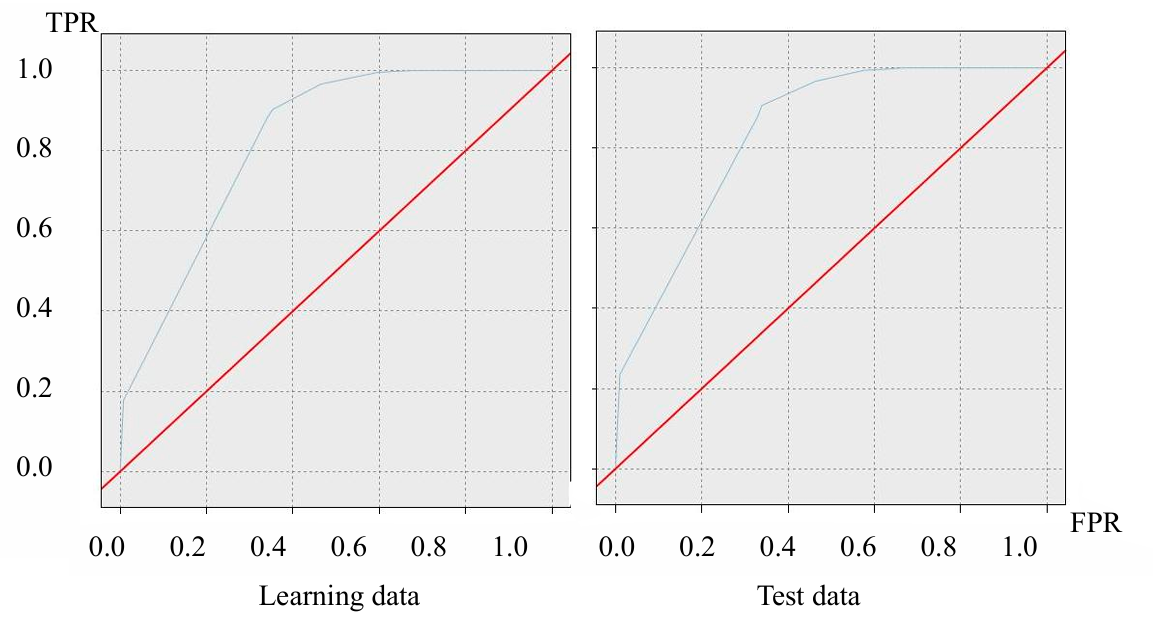
Receiver operating characteristic curve of the created model using the 2022 dataset (7499 cases). Blue line: ROC curve created using the model. FPR: false positive rate; TPR: true positive rate.

The generalization performance of the model was evaluated as follows: the area under the receiver operating characteristic (ROC) curve (AUC) was 0.83 for the training data and 0.84 for the test data. The Gini coefficient for purity of value was 0.65 for the training data and 0.68 for the test data, showing a good performance (Figure 4).

The ROC curve of the created model was generated using the 2022 dataset.

The AUC, which was used as the evaluation index of the model, was close to 1, indicating that the predictive model discriminated accurately. This model is considered useful for discriminating tweets involving individual pharmaceutical transactions.

### Evaluating the Model’s Discriminative Ability

To confirm that the developed model was able to correctly distinguish tweets that may involve individual pharmaceutical product transactions, we used the 2023 dataset to validate the prediction model. The final ensemble model was trained exclusively on the 2022 dataset and was directly applied to the 2023 dataset without retraining, recalibration, or parameter modification. A confusion matrix was constructed using manual annotations of the 2023 dataset as the reference standard (Table 2).

Among 6461 tweets, the model identified 640 true positives and 4678 true negatives, with 158 false positives and 985 false negatives. The overall accuracy was 82.31%. Sensitivity was 39.4%, specificity was 96.73%, the positive predictive value was 80.20%, and the negative predictive value was 82.61%. The relatively high specificity indicates that the model effectively identified tweets not related to individual pharmaceutical transactions, whereas the lower sensitivity suggests that some transaction-related tweets were not detected.

**Figure 5:**
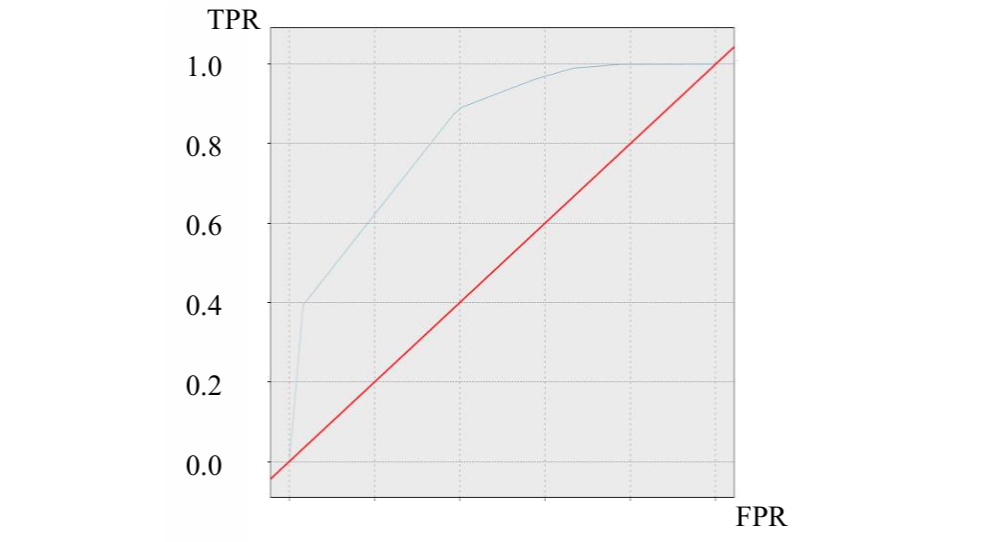
Receiver operating characteristic curve for temporal validation using the 2023 dataset. FPR: false positive rate; TPR: true positive rate.

The model trained on 2022 data was validated using temporally independent 2023 data. The AUC was 0.83 and the Gini coefficient was 0.65, indicating stable discriminative ability. Figure 5 shows the ROC curve for this temporal validation.

**Table 2:** Confusion matrix for model performance on the temporally independent 2023 dataset.

	Predicted suspicious	Predicted nonsuspicious	Total
Manual suspicious	640	985	1625
Manual nonsuspicious	158	4678	4836
Total	798	5663	6461

## Discussion

### Overview

This study demonstrated the feasibility and effectiveness of using machine learning to detect potential individual pharmaceutical transactions on social media. We successfully extracted and ranked keywords appearing frequently in transaction-related tweets and developed a CHAID decision-tree classifier that achieved high accuracy.

### Principal Findings and Keyword Extraction

As a widely used platform for individual pharmaceutical transactions, searches using transaction-related terms always retrieve a large amount of potentially relevant tweets in Twitter, making it difficult to accurately extract associated keywords [25,26]. By leveraging machine learning techniques, we successfully extracted representative keywords from large-scale data to characterize tweets potentially related to individual pharmaceutical transactions. Past studies [27,28] have relied on manual searches and statistical analysis conducted within limited time frames to determine whether content was associated with such activities on Japanese social media platforms [10]. Additionally, one previous study summarized different approaches for social media data collection and sampling [26], such as the development of automated collection and coding platforms [29] and random sampling [30]. However, these approaches often require substantial human resources. Moreover, manual sampling always has a limited duration and scalability of surveillance, and random sampling is limited in terms of general applicability [25,27,30]. Consequently, when the volume of content to be investigated is large, manual searches may be too slow and may fail to capture such activities, because transaction-related tweets can quickly disappear owing to deletion, depletion of available stock, or platform policies. We also found that the closer our search was to the web crawler run date, the more tweets were identified (Figure 2), suggesting that it may be difficult to collect information from SNS tweets because these can be deleted at any time. To detect tweets suspected of involving pharmaceutical transactions between individuals, recent data should be collected on a regular basis to obtain a more accurate picture of the actual situation and extract keywords for use in conducting searches with greater precision. Compared with earlier approaches, our use of a web crawler allowed us to capture traces of tweets existing for only a short period at specific time points. The application of machine learning not only simplifies operations and reduces the need for human resources but also enables fast and accurate extraction of relevant information. This substantially improves detection efficiency and lowers the risk of missing relevant tweets that may be rapidly removed after publication. Integrating web crawlers with keyword-driven classification models thereby facilitates fast, scalable, and automated monitoring of pharmaceutical-related activity on social media platforms.

Notably, unlike English, Japanese text comprises a combination of Hiragana, Katakana, and Kanji, which complicates keyword extraction. Keywords derived from Japanese text segments via text mining do not always form complete words. Therefore, important grammatical or semantic information may be lost. Many extracted keywords, such as #6 “yuzutt,” #27 “Dayviko (*Dayvigo)” (Table S1 in Multimedia Appendix 1), are mere fragments of words. Nevertheless, these exhibited statistically significant associations with tweets suspected of being pharmaceutical transactions. This observation explains the presence of numerous fragmented or incomplete terms (Table 1 and Table S1 in Multimedia Appendix 1), which may appear to lack standalone meaning. This also highlights the unique challenges and potential value of text mining in Japanese-language social media, where important linguistic features are often embedded in partial or colloquial expressions. Despite this limitation, we identified numerous keywords significantly associated with tweets suspected of being individual pharmaceutical transactions. By leveraging these extracted terms, we were able to detect relevant tweets and identify recurring linguistic patterns linked to such activities. Initially, the keyword set was extremely large, making manual searches a cumbersome process. Therefore, we refined the list by focusing on terms with the strongest statistical associations with suspected pharmaceutical transactions. Words such as “DM” (direct message), “kyuu” (request), “yuzuri” (give or transfer), and hashtags like “#Menhera Girl” and “*#Okusuri mogumogu*” were strongly correlated with suspected tweets. The fact that “DM” appeared at the root node of the decision tree underscores the role of private messaging as a key indicator of covert transactional behaviorP (Figure 2).

### Model Performance

The ROC curves for the training dataset (2022) presented in Figure 4 and the temporally independent validation dataset (2023) demonstrated strong discriminative performance, with AUC values of 0.83 for training and 0.84 for testing. The ROC curve for the temporally independent 2023 validation dataset is shown in Figure 5, with an AUC of 0.83 and a Gini coefficient of 0.65, indicating stable predictive performance across temporally separated datasets without evident overfitting. This stability suggests that the modeling framework was able to capture relevant linguistic patterns in the training data. Compared with prior studies using similar datasets or methods, the performance achieved in our study is competitive, thereby supporting the validity of the proposed modeling approach. Additionally, the Gini coefficient of 0.65 for training and 0.68 for testing indicates a clear separation between target classes, reinforcing the model’s practical applicability in real-world scenarios.

In the evaluation of the generated model using the 2023 dataset in this study, 985 (15.25%) tweets related to individual pharmaceutical transactions were incorrectly predicted to be nontransactional (false negatives), and 158 (2.45%) tweets were incorrectly predicted to be suspicious (false positives), as shown in the confusion matrix (Table 2). The ROC curve (Figure 5) also indicated a stable discriminative ability of the model, as evidenced by the curve lying well above the diagonal reference line. These findings suggest that the classifier was able to distinguish between positive and negative classes, although sensitivity was lower than specificity. The overall shape of the curve and its proximity to the top-left corner further support the stability of the model.

These results indicate that although the model achieved satisfactory overall accuracy and the relatively stable AUC values suggest a stable discriminative ability, the relatively low sensitivity indicates that a substantial proportion of transaction-related tweets were not detected, which might be due to the temporal drift between the 2022 and 2023 datasets. Additional evaluation metrics calculated for the temporally independent validation dataset showed an *F*_1_-score of 0.53 and a balanced accuracy of 0.68, suggesting that although the model maintained relatively high specificity and discriminative performance, the lower sensitivity reduced the overall balance of classification performance under moderately imbalanced class distributions. Moreover, the model achieved satisfactory overall accuracy; further improvement is needed, particularly to enhance sensitivity. The fact that our model also extracted words that are not typically included in such tweets suggests that web crawling using an appropriate combination of these keywords may enable more accurate detection of tweets that may be related to individual transactions for pharmaceutical products. Consequently, despite the low sensitivity, the stable AUC value of the model indicates that the model may serve as a prioritization tool for identifying suspicious tweets prior to manual surveillance or platform supervision instead of using a standalone screening tool.

### Cross-Domain Applications and Feasibility of the Multistage Method

Herein, we proposed a multistage method combining web crawling, text mining, and machine learning to detect suspicious information from individual transactions of pharmaceutical products via Twitter. The method features a clear structure and streamlines this process, demonstrating strong applicability for the current research task. Notably, the methods used here have also been widely applied in various research fields and practical settings. Owing to its ability to efficiently capture large volumes of real-time data within a short time frame, web crawling has been applied in finance [31], marketing [32,33], health care [34-36], and medication abuse monitoring [37,38], among others. Leveraging this strength, we could rapidly collect a large dataset of tweets containing information on individual pharmaceutical transactions, from which keywords were extracted for subsequent analysis.

Compared with traditional cross-sectional or prospective surveys, the integration of data mining and machine learning offers several important advantages. These include the capacity to process vast amounts of real-time data, uncover complex patterns without predefined hypotheses, and generate predictive insights. The rapid collection of a large number of tweets substantially reduced the manual workload while providing sufficient data to enhance the accuracy of the machine learning model and support descriptive statistical analyses. Although we primarily used Twitter as the data source, the proposed method is broadly applicable to diverse social networking platforms across different countries, regions, and languages [39]. This not only reflects the technical maturity of the approach, but also reinforces its feasibility and potential for further expansion.

### Limitations

We successfully extracted keywords, as well as emojis, related to individual transactions involving pharmaceutical products. However, our study has several important limitations. First, the analysis focused solely on textual data, excluding visual content such as drug images, quantities, or transaction methods, which often accompany suspected tweets. Consequently, tweets relying primarily on images could not be detected. Second, Twitter’s rebranding to X and the subsequent API changes affected the continuity of data collection in the later stages of the study. Third, owing to platform restrictions and frequent deletion of tweets, the number of recent tweets we were able to collect was limited, affecting the robustness of model validation. Although external validation using data from other social media platforms was not conducted, the model was validated using temporally independent data collected in the subsequent year. Given that language patterns and transaction-related expressions evolve over time, this temporal validation offers preliminary evidence of model robustness within the target platform. Future studies should examine cross-platform applicability to further assess generalizability.

Another limitation is that the web crawler collected tweets using only the hashtag #Okusuri mogumogu. This introduces selection bias, as users engaging in pharmaceutical transactions may deliberately avoid commonly monitored hashtags, meaning that the dataset likely represents only a subset of actual activity. However, during observation and organization of the data collected in this study, we noted that sellers aiming for greater exposure often use multiple hashtags. Continuous monitoring permits timely and effective capture of these transactions as hashtags and keywords are updated. Other hashtags have been documented in previous research, and our approach could be extended to include these; however, in this study, we focused on the single hashtag above to demonstrate the feasibility of the surveillance method. Consequently, the model’s applicability to tweets lacking the monitored hashtag remains untested; future work should explore generalization across additional keywords and platforms. Importantly, the predictive model did not include the monitored hashtag itself as a feature but was trained on linguistic patterns and statistically significant keywords derived from text mining. Therefore, the classification was based on transactional language characteristics rather than the presence of a specific hashtag.

It should be noted that the current model primarily emphasized specificity, and a limited exploration of classification threshold adjustment may have contributed to low sensitivity during observation.

Despite these limitations, the model’s consistent performance across datasets underscores its practical potential in digital surveillance. This approach may provide a useful methodological foundation for future regulatory frameworks aimed at identifying and intervening in illegal pharmaceutical transactions on social networking platforms. Additionally, studies incorporating multiple hashtags and keyword-based crawling approaches may further improve the robustness and practical applicability of the proposed model in the future.

### Conclusions

By identifying transaction search tags and using manual searches to supplement web crawling and text mining, together with machine learning, we were able to identify keywords related to individual transactions involving pharmaceutical products and extract characteristic words to successfully create a prediction model. This method of identifying keywords from tweets involving pharmaceuticals suspected of being privately trafficked could help to prevent individual transactions involving pharmaceutical products.

## Appendix

Multimedia Appendix 1 List of extracted and statistically significant words from the 2022 collected postings.

## Abbreviations

API: application programming interface
AUC: area under the curve
CHAID: chi-square automatic interaction detection
ROC: receiver operating characteristic
SNS: social networking service

## Data Availability

The datasets are not publicly available owing to the Ministry of Health, Labor and Welfare. Requests can be made to the corresponding author for academic purposes.
