# Supplementary material for: Methods for Detecting Suspicious Information From Individual Transactions of Pharmaceutical Products via Twitter (now X): Retrospective Observational Study
Source: J Med Internet Res. 2026 Jun 19;28:e91103. doi: 10.2196/91103 (PMC13332370; doi:10.2196/91103)
Supplement: Multimedia Appendix 1 [file jmir_v28i1e91103_app1.docx]

Table S1. List of extracted and statistically significant words from the 2022 collected postings.

| No. | Extracted words | Total number of posts that the word appeared =【α】 | Total number of posts in which the word appears among posts with suspected transactions =【β】 | Percentage of 【β】/【α】 |
| --- | --- | --- | --- | --- |
| 1 | kyuu | 83 | 81 | 97.59% |
| 2 | Yuzuri | 103 | 100 | 97.09% |
| 3 | Pushcart | 28 | 27 | 96.43% |
| 4 | receive | 25 | 24 | 96.00% |
| 5 | profile | 23 | 22 | 95.65% |
| 6 | yuzutt | 131 | 125 | 95.42% |
| 7 | List | 42 | 40 | 95.24% |
| 8 | customary | 21 | 20 | 95.24% |
| 9 | enlightenment | 20 | 19 | 95.00% |
| 10 | requestion | 19 | 18 | 94.74% |
| 11 | various | 30 | 28 | 93.33% |
| 12 | hope | 53 | 49 | 92.45% |
| 13 | zol | 12 | 11 | 91.67% |
| 14 | reference | 23 | 21 | 91.30% |
| 15 | questionnaire | 11 | 10 | 90.91% |
| 16 | cheaping | 70 | 63 | 90.00% |
| 17 | @tos | 40 | 36 | 90.00% |
| 18 | financial amount | 20 | 18 | 90.00% |
| 19 | could get | 20 | 18 | 90.00% |
| 20 | Postal Service | 10 | 9 | 90.00% |
| 21 | 25 | 10 | 9 | 90.00% |
| 22 | Twi-profi (*Twitter profile) | 10 | 9 | 90.00% |
| 23 | URL | 39 | 35 | 89.74% |
| 24 | addition | 29 | 26 | 89.66% |
| 25 | dm | 19 | 17 | 89.47% |
| 26 | 5st | 18 | 16 | 88.89% |
| 27 | Dayviko (*Dayvigo) | 18 | 16 | 88.89% |
| 28 | Please give | 9 | 8 | 88.89% |
| 29 | other name | 9 | 8 | 88.89% |
| 30 | Lemborexant | 9 | 8 | 88.89% |
| 31 | bachibachidown | 9 | 8 | 88.89% |
| 32 | bureau | 9 | 8 | 88.89% |
| 33 | chilled | 9 | 8 | 88.89% |
| 34 | keep ...-ing | 9 | 8 | 88.89% |
| 35 | pleas(*Please) | 9 | 8 | 88.89% |
| 36 | d | 9 | 8 | 88.89% |
| 37 | s10 | 9 | 8 | 88.89% |
| 38 | subscription | 9 | 8 | 88.89% |
| 39 | find | 74 | 65 | 87.84% |
| 40 | fixa (*Fixation) | 40 | 35 | 87.50% |
| 41 | pa | 16 | 14 | 87.50% |
| 42 | 2m | 8 | 7 | 87.50% |
| 43 | discount | 8 | 7 | 87.50% |
| 44 | how much | 8 | 7 | 87.50% |
| 45 | sorry | 8 | 7 | 87.50% |
| 46 | personal delivery | 8 | 7 | 87.50% |
| 47 | buy | 8 | 7 | 87.50% |
| 48 | anonymity | 8 | 7 | 87.50% |
| 49 | @grenn_420 | 8 | 7 | 87.50% |
| 50 | deadline | 8 | 7 | 87.50% |
| 51 | @aunch1823 | 8 | 7 | 87.50% |
| 52 | #UNCH | 8 | 7 | 87.50% |
| 53 | to request | 39 | 34 | 87.18% |
| 54 | DM | 381 | 332 | 87.14% |
| 55 | #LUNESTA | 30 | 26 | 86.67% |
| 56 | low price | 15 | 13 | 86.67% |
| 57 | #CONTOMIN | 14 | 12 | 85.71% |
| 58 | 125 | 7 | 6 | 85.71% |
| 59 | ly | 7 | 6 | 85.71% |
| 60 | depends on | 7 | 6 | 85.71% |
| 61 | @null | 7 | 6 | 85.71% |
| 62 | update | 48 | 41 | 85.42% |
| 63 | uses | 27 | 23 | 85.19% |
| 64 | exchange | 20 | 17 | 85.00% |
| 65 | need | 20 | 17 | 85.00% |
| 66 | #Etizolam | 32 | 27 | 84.38% |
| 67 | stock | 48 | 40 | 83.33% |
| 68 | above | 24 | 20 | 83.33% |
| 69 | Here you go | 24 | 20 | 83.33% |
| 70 | ora (*Parts of Japanese Honorifics) | 12 | 10 | 83.33% |
| 71 | remaining | 12 | 10 | 83.33% |
| 72 | Huri (*First half of the word payment via bank in Japanese) | 6 | 5 | 83.33% |
| 73 | HURI (*First half of the word payment via bank in Japanese) | 6 | 5 | 83.33% |
| 74 | R | 6 | 5 | 83.33% |
| 75 | Pei(*Pay in Japanese) | 6 | 5 | 83.33% |
| 76 | 〇 | 6 | 5 | 83.33% |
| 77 | garnish | 6 | 5 | 83.33% |
| 78 | Gene (*Generic) | 6 | 5 | 83.33% |
| 79 | 7000 | 6 | 5 | 83.33% |
| 80 | GIRE | 6 | 5 | 83.33% |
| 81 | nao | 6 | 5 | 83.33% |
| 82 | seek | 6 | 5 | 83.33% |
| 83 | @420_grenn | 6 | 5 | 83.33% |
| 84 | 2seat | 6 | 5 | 83.33% |
| 85 | citation | 6 | 5 | 83.33% |
| 86 | appeal | 6 | 5 | 83.33% |
| 87 | UP | 6 | 5 | 83.33% |
| 88 | open | 6 | 5 | 83.33% |
| 89 | #MIYAKAWAHIBIKI | 6 | 5 | 83.33% |
| 90 | 1mg | 47 | 39 | 82.98% |
| 91 | Please give | 29 | 24 | 82.76% |
| 92 | ETI | 23 | 19 | 82.61% |
| 93 | #LENDORMIN | 11 | 9 | 81.82% |
| 94 | summary | 11 | 9 | 81.82% |
| 95 | @1 | 11 | 9 | 81.82% |
| 96 | finished | 32 | 26 | 81.25% |
| 97 | book | 31 | 25 | 80.65% |
| 98 | lorazepam | 20 | 16 | 80.00% |
| 99 | skusyo (*The abbreviation of screenshot in Japanese) | 15 | 12 | 80.00% |
| 100 | itashi | 10 | 8 | 80.00% |
| 101 | remai (*Remain) | 157 | 124 | 78.98% |
| 102 | arrival | 19 | 15 | 78.95% |
| 103 | Din (*EURODIN) | 14 | 11 | 78.57% |
| 104 | who | 14 | 11 | 78.57% |
| 105 | status quo | 14 | 11 | 78.57% |
| 106 | generic | 41 | 32 | 78.05% |
| 107 | price | 41 | 32 | 78.05% |
| 108 | 150 | 9 | 7 | 77.78% |
| 109 | shipping | 9 | 7 | 77.78% |
| 110 | free market | 71 | 55 | 77.46% |
| 111 | EURO (*EURODIN) | 13 | 10 | 76.92% |
| 112 | total | 13 | 10 | 76.92% |
| 113 | or | 17 | 13 | 76.47% |
| 114 | osaka | 17 | 13 | 76.47% |
| 115 | confirmation | 58 | 44 | 75.86% |
| 116 | Lunesta | 61 | 46 | 75.41% |
| 117 | 2mg | 48 | 36 | 75.00% |
| 118 | details | 16 | 12 | 75.00% |
| 119 | in stock | 16 | 12 | 75.00% |
| 120 | no matter what | 16 | 12 | 75.00% |
| 121 | recognition | 12 | 9 | 75.00% |
| 122 | Estazolam | 8 | 6 | 75.00% |
| 123 | lottery | 8 | 6 | 75.00% |
| 124 | 800 | 8 | 6 | 75.00% |
| 125 | xo (*Loxonin) | 8 | 6 | 75.00% |
| 126 | flunit (*Flunitrazepam) | 8 | 6 | 75.00% |
| 127 | last day | 8 | 6 | 75.00% |
| 128 | triazolam | 8 | 6 | 75.00% |
| 129 | #okusuri | 8 | 6 | 75.00% |
| 130 | original | 8 | 6 | 75.00% |
| 131 | being gone | 8 | 6 | 75.00% |
| 132 | tele | 99 | 74 | 74.75% |
| 133 | RAVONA | 35 | 26 | 74.29% |
| 134 | 5mg | 31 | 23 | 74.19% |
| 135 | flunitrazepam | 58 | 43 | 74.14% |
| 136 | free market app | 27 | 20 | 74.07% |
| 137 | silver | 23 | 17 | 73.91% |
| 138 | remainder | 19 | 14 | 73.68% |
| 139 | helping | 19 | 14 | 73.68% |
| 140 | waiting | 30 | 22 | 73.33% |
| 141 | 50 | 15 | 11 | 73.33% |
| 142 | 2st | 15 | 11 | 73.33% |
| 143 | only | 44 | 32 | 72.73% |
| 144 | 75 | 11 | 8 | 72.73% |
| 145 | CONTO (*CONTOMIN) | 11 | 8 | 72.73% |
| 146 | take | 11 | 8 | 72.73% |
| 147 | #Dayvigo | 40 | 29 | 72.50% |
| 148 | PayPay | 47 | 34 | 72.34% |
| 149 | 1000 | 18 | 13 | 72.22% |
| 150 | nite (*) | 42 | 30 | 71.43% |
| 151 | # RAVONA | 35 | 25 | 71.43% |
| 152 | sale | 35 | 25 | 71.43% |
| 153 | kata (*Past tense verb in Japanese) | 14 | 10 | 71.43% |
| 154 | #Zyprexa | 14 | 10 | 71.43% |
| 155 | bonus | 7 | 5 | 71.43% |
| 156 | 100mg | 7 | 5 | 71.43% |
| 157 | Can sale | 7 | 5 | 71.43% |
| 158 | #thco | 7 | 5 | 71.43% |
| 159 | trust | 7 | 5 | 71.43% |
| 160 | disclosure | 7 | 5 | 71.43% |
| 161 | m | 17 | 12 | 70.59% |
| 162 | Etizolam | 50 | 35 | 70.00% |
| 163 | Benzarin | 20 | 14 | 70.00% |
| 164 | low price | 10 | 7 | 70.00% |
| 165 | Levotomin | 10 | 7 | 70.00% |
| 166 | 75mg | 10 | 7 | 70.00% |
| 167 | 18 | 10 | 7 | 70.00% |
| 168 | remainin (*Remaining) | 10 | 7 | 70.00% |
| 169 | fixed-term | 29 | 20 | 68.97% |
| 170 | give | 151 | 103 | 68.21% |
| 171 | #Hirnamin | 22 | 15 | 68.18% |
| 172 | blank | 22 | 15 | 68.18% |
| 173 | DEADLINE | 22 | 15 | 68.18% |
| 174 | correspondence | 47 | 32 | 68.09% |
| 175 | want | 53 | 36 | 67.92% |
| 176 | twi | 59 | 40 | 67.80% |
| 177 | Please | 197 | 133 | 67.51% |
| 178 | #Zolpidem | 45 | 30 | 66.67% |
| 179 | present | 42 | 28 | 66.67% |
| 180 | others | 24 | 16 | 66.67% |
| 181 | thing | 12 | 8 | 66.67% |
| 182 | 1s | 12 | 8 | 66.67% |
| 183 | past results | 12 | 8 | 66.67% |
| 184 | in detail | 12 | 8 | 66.67% |
| 185 | Cymbalta | 9 | 6 | 66.67% |
| 186 | #Antidepressant | 9 | 6 | 66.67% |
| 187 | pa | 23 | 15 | 65.22% |
| 188 | Gozai (*Parts of Japanese Honorifics) | 77 | 50 | 64.94% |
| 189 | inquiry | 17 | 11 | 64.71% |
| 190 | report | 45 | 29 | 64.44% |
| 191 | paypay | 25 | 16 | 64.00% |
| 192 | Buy (*Different grammar with No.47 in Japanese) | 47 | 30 | 63.83% |
| 193 | Sending | 58 | 37 | 63.79% |
| 194 | 10 | 80 | 51 | 63.75% |
| 195 | #Quetiapine | 11 | 7 | 63.64% |
| 196 | #Pregabalin | 11 | 7 | 63.64% |
| 197 | Please give me | 182 | 115 | 63.19% |
| 198 | welcome | 19 | 12 | 63.16% |
| 199 | sheet | 100 | 63 | 63.00% |
| 200 | ♂ | 27 | 17 | 62.96% |
| 201 | All of | 27 | 17 | 62.96% |
| 202 | activity | 35 | 22 | 62.86% |
| 203 | Ori (*Parts of Japanese Honorifics) | 35 | 22 | 62.86% |
| 204 | with the exception of | 43 | 27 | 62.79% |
| 205 | remain | 16 | 10 | 62.50% |
| 206 | It's okay | 61 | 38 | 62.30% |
| 207 | Exi(*Exist) | 42 | 26 | 61.90% |
| 208 | Flu (*Flunitrazepam) | 26 | 16 | 61.54% |
| 209 | #DM please | 13 | 8 | 61.54% |
| 210 | Halcion | 13 | 8 | 61.54% |
| 211 | 20mg | 13 | 8 | 61.54% |
| 212 | Depa (*Depas) | 36 | 22 | 61.11% |
| 213 | #Flunitrazepam | 58 | 35 | 60.34% |
| 214 | cheap | 30 | 18 | 60.00% |
| 215 | min | 20 | 12 | 60.00% |
| 216 | degree | 15 | 9 | 60.00% |
| 217 | #I will give you the medicine | 254 | 152 | 59.84% |
| 218 | desired | 184 | 110 | 59.78% |
| 219 | ira (*Parts of WELCOME in Japanese) | 32 | 19 | 59.38% |
| 220 | box | 22 | 13 | 59.09% |
| 221 | application | 12 | 7 | 58.33% |
| 222 | Received (*In Japanese Honorifics) | 12 | 7 | 58.33% |
| 223 | Well (*In Japanese Honorifics) | 12 | 7 | 58.33% |
| 224 | early | 12 | 7 | 58.33% |
| 225 | Sile (*Silece) | 12 | 7 | 58.33% |
| 226 | de(*Depas) | 248 | 144 | 58.06% |
| 227 | # sleeping pills | 69 | 40 | 57.97% |
| 228 | pregabalin | 19 | 11 | 57.89% |
| 229 | milli (*Milligram) | 47 | 27 | 57.45% |
| 230 | Merkari (*A free market site/app in Japan) | 47 | 27 | 57.45% |
| 231 | zolpidem | 35 | 20 | 57.14% |
| 232 | Solanax | 28 | 16 | 57.14% |
| 233 | Depakene | 14 | 8 | 57.14% |
| 234 | 300 | 14 | 8 | 57.14% |
| 235 | possible | 62 | 35 | 56.45% |
| 236 | pas (*Depas) | 224 | 126 | 56.25% |
| 237 | 25mg | 16 | 9 | 56.25% |
| 238 | send | 16 | 9 | 56.25% |
| 239 | # stabilizer | 16 | 9 | 56.25% |
| 240 | Wypax | 27 | 15 | 55.56% |
| 241 | Dayvigo | 47 | 26 | 55.32% |
| 242 | contents | 20 | 11 | 55.00% |
| 243 | itasi (*Parts of Japanese Honorifics) | 51 | 28 | 54.90% |
| 244 | Lece (*Silece) | 236 | 129 | 54.66% |
| 245 | Concerta | 86 | 47 | 54.65% |
| 246 | Si (*Silece) | 291 | 158 | 54.30% |
| 247 | plan | 65 | 35 | 53.85% |
| 248 | packaging | 26 | 14 | 53.85% |
| 249 | can be made | 28 | 15 | 53.57% |
| 250 | large quantity | 60 | 32 | 53.33% |
| 251 | Quetiapine | 32 | 17 | 53.13% |
| 252 | 10mg | 36 | 19 | 52.78% |
| 253 | 5 | 21 | 11 | 52.38% |
| 254 | fixation | 23 | 12 | 52.17% |
| 255 | wai (*Waiting) | 25 | 13 | 52.00% |
| 256 | profi (*Profile) | 66 | 33 | 50.00% |
| 257 | Thanks | 44 | 22 | 50.00% |
| 258 | #RIVOTRIL | 32 | 16 | 50.00% |
| 259 | pay attention to | 32 | 16 | 50.00% |
| 260 | Lendormin | 22 | 11 | 50.00% |
| 261 | #Rohypnol | 20 | 10 | 50.00% |
| 262 | please | 514 | 254 | 49.42% |
| 263 | lyrica | 76 | 37 | 48.68% |
| 264 | Writi Writing | 29 | 14 | 48.28% |
| 265 | stuc (*Stuck) | 25 | 12 | 48.00% |
| 266 | transactions | 111 | 53 | 47.75% |
| 267 | Lexotan | 49 | 23 | 46.94% |
| 268 | #lyrica | 47 | 22 | 46.81% |
| 269 | Belsomra | 28 | 13 | 46.43% |
| 270 | Letterpa (*Letter Pack) | 39 | 18 | 46.15% |
| 271 | considerably | 26 | 12 | 46.15% |
| 272 | ease | 42 | 19 | 45.24% |
| 273 | best regards | 89 | 40 | 44.94% |
| 274 | ♀ | 65 | 29 | 44.62% |
| 275 | 1st | 46 | 20 | 43.48% |
| 276 | bu (*buy) | 37 | 16 | 43.24% |
| 277 | 20 | 44 | 19 | 43.18% |
| 278 | sleep | 124 | 53 | 42.74% |
| 279 | can | 82 | 35 | 42.68% |
| 280 | lee (*Myslee) | 203 | 86 | 42.36% |
| 281 | gather together | 45 | 19 | 42.22% |
| 282 | RIVOTRIL | 39 | 16 | 41.03% |
| 283 | agent (*From sleeping agent) | 151 | 61 | 40.40% |
| 284 | #Silece | 274 | 106 | 38.69% |
| 285 | go (*Parts of Japanese Honorifics) | 218 | 84 | 38.53% |
| 286 | 2 | 137 | 52 | 37.96% |
| 287 | #okusurimogumogu | 162 | 60 | 37.04% |
| 288 | talking | 102 | 37 | 36.27% |
| 289 | Contacts | 292 | 100 | 34.25% |
| 290 | 1 | 214 | 73 | 34.11% |
| 291 | #DEPAS | 497 | 144 | 28.97% |
| 292 | #Myslee | 334 | 95 | 28.44% |
| 293 | #Okusuri mogumogu | 6682 | 1641 | 24.56% |
| 294 | ing (*Parts of verb-ing in Japanese) | 737 | 142 | 19.27% |
| 295 | tara (*If in Japanese sentense) | 747 | 127 | 17.00% |
| 296 | kedo (*But in Japanese sentense) | 456 | 74 | 16.23% |
| 297 | re | 478 | 75 | 15.69% |
| 298 | mogumogu | 370 | 57 | 15.41% |
| 299 | ki | 210 | 32 | 15.24% |
| 300 | AKA(*Parts of Account in Japanese) | 278 | 42 | 15.11% |
| 301 | ask | 7 | 1 | 14.29% |
| 302 | ba | 316 | 45 | 14.24% |
| 303 | tsuke (*Parts of Add in or Be careful in Japanese) | 142 | 18 | 12.68% |
| 304 | tomorrow | 136 | 17 | 12.50% |
| 305 | today | 215 | 26 | 12.09% |
| 306 | information | 100 | 12 | 12.00% |
| 307 | daro (*Parts of Maybe in Japanese) | 78 | 9 | 11.54% |
| 308 | er (*Parts of Fraudster in Japanese) | 128 | 14 | 10.94% |
| 309 | DE (*be used for DEPAS in Japanese Kanji, also can be a part of sending out) | 76 | 8 | 10.53% |
| 310 | Na (*Parts of become in Japanese) | 232 | 23 | 9.91% |
| 311 | kamo (*Parts of Maybe in Japanese) | 92 | 9 | 9.78% |
| 312 | Naa (*A tone of inflection) | 175 | 17 | 9.71% |
| 313 | be too | 83 | 8 | 9.64% |
| 314 | Or | 64 | 6 | 9.38% |
| 315 | #Lexotan | 161 | 15 | 9.32% |
| 316 | Prof (*Profile) | 451 | 39 | 8.65% |
| 317 | Friends | 70 | 6 | 8.57% |
| 318 | BURON (*A Japanese antitussive product named S-Swiss Bron) | 72 | 6 | 8.33% |
| 319 | got | 75 | 6 | 8.00% |
| 320 | Tyotto (*Just/A little in Japanese) | 50 | 4 | 8.00% |
| 321 | Account | 76 | 6 | 7.89% |
| 322 | #Mental Illness | 51 | 4 | 7.84% |
| 323 | Increase | 52 | 4 | 7.69% |
| 324 | Siyo (*Parts of What can I do or Let’s do in Japanese) | 67 | 5 | 7.46% |
| 325 | Nomi (*Take of take medicine in Japanese) | 124 | 9 | 7.26% |
| 326 | Amount | 42 | 3 | 7.14% |
| 327 | Prescription notes | 28 | 2 | 7.14% |
| 328 | Nomu (*Take of take medicine in Japanese) | 72 | 5 | 6.94% |
| 329 | Message | 234 | 16 | 6.84% |
| 330 | Hospital | 105 | 7 | 6.67% |
| 331 | Everyone | 45 | 3 | 6.67% |
| 332 | Fluffy | 46 | 3 | 6.52% |
| 333 | immediately | 46 | 3 | 6.52% |
| 334 | Add | 77 | 5 | 6.49% |
| 335 | RT | 2037 | 132 | 6.48% |
| 336 | Use | 49 | 3 | 6.12% |
| 337 | Limited | 67 | 4 | 5.97% |
| 338 | Head | 34 | 2 | 5.88% |
| 339 | Feeling | 34 | 2 | 5.88% |
| 340 | LINE | 52 | 3 | 5.77% |
| 341 | #Fraudster | 52 | 3 | 5.77% |
| 342 | While | 70 | 4 | 5.71% |
| 343 | KA (*Department of psychiatry in Japanese) | 35 | 2 | 5.71% |
| 344 | #OD | 263 | 15 | 5.70% |
| 345 | Twitter | 107 | 6 | 5.61% |
| 346 | Overseas | 54 | 3 | 5.56% |
| 347 | Introduction | 54 | 3 | 5.56% |
| 348 | Good | 92 | 5 | 5.43% |
| 349 | Tokyo | 37 | 2 | 5.41% |
| 350 | Attention | 351 | 18 | 5.13% |
| 351 | Aimed at | 39 | 2 | 5.13% |
| 352 | Such | 80 | 4 | 5.00% |
| 353 | Sleep | 40 | 2 | 5.00% |
| 354 | Live | 40 | 2 | 5.00% |
| 355 | Think | 40 | 2 | 5.00% |
| 356 | Thinking | 370 | 18 | 4.86% |
| 357 | Thinkin (*Thinking) | 62 | 3 | 4.84% |
| 358 | be able (*Parts of be able to do in Japanese) | 444 | 21 | 4.73% |
| 359 | #YAMIAKA (*Sick Account in Japanese) | 127 | 6 | 4.72% |
| 360 | Non (*Take of take medicine in Japanese) | 234 | 11 | 4.70% |
| 361 | Problem | 48 | 2 | 4.17% |
| 362 | #BURON (*A Japanese antitussive product named S-Swiss Bron) | 235 | 9 | 3.83% |
| 363 | 24 | 210 | 8 | 3.81% |
| 364 | Harm | 106 | 4 | 3.77% |
| 365 | Individual | 213 | 8 | 3.76% |
| 366 | Himself/Herself | 27 | 1 | 3.70% |
| 367 | #Medicon | 164 | 6 | 3.66% |
| 368 | Selling | 83 | 3 | 3.61% |
| 369 | Live Stream | 28 | 1 | 3.57% |
| 370 | MENHERA (*People who are mentally unhealthy in Japanese.) | 28 | 1 | 3.57% |
| 371 | Transfer | 28 | 1 | 3.57% |
| 372 | Popularity | 85 | 3 | 3.53% |
| 373 | Together | 29 | 1 | 3.45% |
| 374 | Kuri (*Parts of Christmas in Japanese) | 30 | 1 | 3.33% |
| 375 | OD | 363 | 12 | 3.31% |
| 376 | Masyo (*Parts of Let’s Together in Japanese) | 122 | 4 | 3.28% |
| 377 | Transmit | 31 | 1 | 3.23% |
| 378 | men | 32 | 1 | 3.13% |
| 379 | Japan | 65 | 2 | 3.08% |
| 380 | go | 99 | 3 | 3.03% |
| 381 | Rely on | 66 | 2 | 3.03% |
| 382 | #Hope To Spreading | 33 | 1 | 3.03% |
| 383 | NO | 33 | 1 | 3.03% |
| 384 | This week | 34 | 1 | 2.94% |
| 385 | Prescription | 419 | 12 | 2.86% |
| 386 | Risk | 36 | 1 | 2.78% |
| 387 | Prevention | 38 | 1 | 2.63% |
| 388 | Half-way | 38 | 1 | 2.63% |
| 389 | Good terms with | 39 | 1 | 2.56% |
| 390 | Bad | 39 | 1 | 2.56% |
| 391 | Evening | 353 | 9 | 2.55% |
| 392 | #YAMIAKASANTOTSUNAGARITAI (*I Want To Connect With Sick Account Users in Japanese) | 503 | 12 | 2.39% |
| 393 | getting up | 42 | 1 | 2.38% |
| 394 | as | 42 | 1 | 2.38% |
| 395 | procedure | 42 | 1 | 2.38% |
| 396 | Illegal | 86 | 2 | 2.33% |
| 397 | about | 132 | 3 | 2.27% |
| 398 | Morning | 132 | 3 | 2.27% |
| 399 | Akasi (*Parts of Evidence in Japanese) | 44 | 1 | 2.27% |
| 400 | hara (*Parts of Pay in Japanese) | 44 | 1 | 2.27% |
| 401 | Awakening (*Parts of Stimulant Agents in Japanese) | 45 | 1 | 2.22% |
| 402 | Buying and Selling | 45 | 1 | 2.22% |
| 403 | Uwa (*Parts of That's Why or | 45 | 1 | 2.22% |
| 404 | Phoning in Japanese) | 46 | 1 | 2.17% |
| 405 | #Psychiatry | 331 | 7 | 2.11% |
| 406 | Wholly | 99 | 2 | 2.02% |
| 407 | Job | 50 | 1 | 2.00% |
| 408 | Harsh | 51 | 1 | 1.96% |
| 409 | Insurance | 103 | 2 | 1.94% |
| 410 | Uke (*Parts of Accept in Japanese) | 103 | 2 | 1.94% |
| 411 | rareru (*Parts of Japanese Honorifics or meaning possibility in Japanese) | 208 | 4 | 1.92% |
| 412 | reminder | 55 | 1 | 1.82% |
| 413 | #YAMIAKAJYOSI (Sick Account User is A Girl in Japanese) | 234 | 4 | 1.71% |
| 414 | By all means (Parts of Japanese Honorifics) | 61 | 1 | 1.64% |
| 415 | #Pharmaceuticals | 187 | 3 | 1.60% |
| 416 | reply | 259 | 4 | 1.54% |
| 417 | OK | 131 | 2 | 1.53% |
| 418 | Insomnia | 333 | 5 | 1.50% |
| 419 | Management | 67 | 1 | 1.49% |
| 420 | #Stimulant Agents | 71 | 1 | 1.41% |
| 421 | Penal Servitude | 234 | 3 | 1.28% |
| 422 | Disease | 78 | 1 | 1.28% |
| 423 | Drugstore | 314 | 4 | 1.27% |
| 424 | Especially | 161 | 2 | 1.24% |
| 425 | Notice | 84 | 1 | 1.19% |
| 426 | Misuse | 172 | 2 | 1.16% |
| 427 | Import | 101 | 1 | 0.99% |
| 428 | suspicious | 101 | 1 | 0.99% |
| 429 | PHARMACEUTICALS | 109 | 1 | 0.92% |
| 430 | net | 773 | 7 | 0.91% |
| 431 | hairi (*Parts of In in Japanese) | 114 | 1 | 0.88% |
| 432 | #Individual Import | 115 | 1 | 0.87% |
| 433 | homepage | 121 | 1 | 0.83% |
| 434 | #Cannabis | 858 | 7 | 0.82% |
| 435 | #MENHERA (*People who are mentally unhealthy in Japanese.) | 517 | 4 | 0.77% |
| 436 | mentality | 397 | 3 | 0.76% |
| 437 | medical care | 811 | 6 | 0.74% |
| 438 | haire (*Parts of In in Japanese) | 313 | 2 | 0.64% |
| 439 | reality | 318 | 2 | 0.63% |
| 440 | ea (*Parts of eat in Japanese) | 165 | 1 | 0.61% |
| 441 | #DXM | 165 | 1 | 0.61% |
| 442 | pharmaceuticals | 396 | 2 | 0.51% |
| 443 | link | 420 | 2 | 0.48% |
| 444 | tag | 801 | 3 | 0.37% |
| 445 | #MENHERA to tsunagaritai (*Want to connect with people who are mentally unhealthy in Japanese.) | 325 | 1 | 0.31% |
| 446 | let alone | 329 | 1 | 0.30% |
| 447 | resale | 349 | 1 | 0.29% |
| 448 | hell | 359 | 1 | 0.28% |
| 449 | # Sleeping Disorders | 370 | 1 | 0.27% |
| 450 | KUSURI (*Drugs in Japanese, also can from Okusurimogumogu) | 424 | 1 | 0.24% |
| 451 | #overdose | 445 | 1 | 0.22% |
| 452 | KOU (*The first Kanji for Psychotropics in Japanese) | 796 | 1 | 0.13% |

・452 words in total, sorted by Total number of posts in which the word appears among posts with suspected transactions=【β】/ Total number of posts that the word appeared =【α】, in descending order, the p-values <0.01 each of all the words.

・The black squares are accounts and information that cannot be disclosed.

・In the translation process, we have the Hiragana, Katakana, Kanji and English case correspondences. Since all three expressions are recognized as different characters in Japanese, and thus recognized as different keywords by the text mining program we use, the same word appears several times in the table, just for case differentiation.

・We have also added the split English words where the Japanese characters were split, and we have added the original words that may have been split in the (*) section as an annotation reference.

・Kanji characters are uppercased first, katakana characters are all uppercased, hiragana characters are lowercase, and English characters are extracted as they are.
